# Supplementary material for: Connecting higher‐order interactions with ecological stability in experimental aquatic food webs
Source: Ecol Evol. 2023 Sep 6;13(9):e10502. doi: 10.1002/ece3.10502 (PMC10483096; doi:10.1002/ece3.10502)

# Supplementary material

**S1. Preparation of bacterial cultures**

One week before the start of the community experiment, we prepared the bacterial medium for prey cultures. We first added 3-4mL DMSZ medium into three 20mL Falcon tubes, then transferred *B. subtilis, B. brevis* and *S. fonticola* to these tubes using inoculation loops and incubated them at 37°C for 24 hours.

After one day of incubation, we added 100µl of *S. fonticola* and *B. subtilis* and 2ml of *B. brevis* medium (since *B. brevis* grows much slower than *S. fonticola* and *B. subtilis*) from the Falcon tubes to three previously autoclaved 1L glass bottles, each containing 500ml of ciliates pellet medium. We then incubated them at 20°C for two days to give the bacteria enough time to reach their carrying capacity. The prey culture medium was produced by mixing 33.3ml of each bacteria medium from the 1L glass bottles into an autoclaved 250 mL jar (i.e., the microcosm), yielding a total of 100ml of mixed bacterial culture medium. Two wheat seeds were added to each jar to provide a source of slow release nutrients that the bacteria could feed on, leading to stable populations of ciliates during our experiment (Altermatt et al., 2015). All the consumers were grown at 15° C prior to the experiments for three weeks to reach their carrying capacities.

**S2. Species classification**

For species classification, we used the videos collected from the first sampling day of every week and randomly chose one video of each replicate for the six community compositions. By observing the morphology and movement patterns of the ciliates in the videos, we labelled 350 to 400 trajectories of each species as training data. We use a similar number of labelled trajectories of each species, which avoids low classification performance for rare classes (e.g. low abundance species) caused by imbalanced numbers of observations (Sommer & Gerlich, 2013). Although the trajectory filtering function from BEMOVI removed most of the background noise, the movements of some tiny, suspended impurities may remain as spurious trajectories within the training data. In order to prevent these trajectories from being mistakenly classified as other species, we selected 100 videos that we visually checked to contain no ciliates and labelled all detected trajectories as “noise”. Trajectories of class “noise” were then excluded from all subsequent analyses.

Twenty features extracted from an established classification pipeline (Pennekamp et al., 2017) were selected to train the classification algorithm to distinguish among classes based on information about body size and movement patterns (Table S1). As the phenotypes of ciliates may change over time (Pennekamp et al., 2017), the week number was added as the 21^st^ feature in the classification algorithm to enhance the accuracy of the prediction.

As the species included in each treatment were different, we built six customized models for classification that only contained the known species of each community. Prior to the experiment, we compared the random forest (RF) and Support Vector Machine (SVM) as our classifiers for their computation efficiency and reliability of results (Fernández-Delgado et al., 2014). We used the randomForest package (Liaw & Wiener, 2002) and e1071 packages that contain the *svm* function (Meyer et al., 2014.). We compared the performances of two classifiers by calculating the classification errors for all training data (Table S2). Since SVM gave us overall lower classification errors, we applied it to our six customized models and generated time series of change density change of species for each community.

**Table S1:** Morphological and movement features selected for use in classification

| Code | Measurement method |
| --- | --- |
| mean_area | Mean area of particle across trajectory |
| sd_area | Standard deviation of particle area |
| mean_perimeter | Mean length of perimeter of particle |
| sd_perimeter | Standard deviation of particle perimeter length |
| mean_major | Mean length of major axis of ellipse fitted to particle |
| sd_major | Standard deviation of length of major axis |
| mean_ar | Mean aspect ratio of particle |
| sd_ar | Standard deviation of particle aspect ratio |
| mean_turning | Numeric mean of particle direction |
| sd_turning | Circular standard deviation of particle direction |
| gross_disp | Gross displacement (sum of all steps of a trajectory) |
| max_net | Maximum net displacement |
| net_disp | Net displacement |
| net_speed | Net displacement travelled between frames |
| max_step | Maximum step length |
| min_step | Minimum step length |
| sd_step | Standard deviation of step length |
| sd_gross_speed | Standard deviation of distance travelled between frames |
| max_gross_speed | Maximum distance travelled between frames |
| min_gross_speed | Minimum distance travelled between frames |

**Table S2:** Comparison of the model performances between Random Forest and Support Vector Machine

| Predicted  True | Colp | Dexio | Noise | Para | Spath | Spiro | Class.error |
| --- | --- | --- | --- | --- | --- | --- | --- |
|  | RandomForest | | | | | | |
| Colp | 347 | 19 | 0 | 12 | 9 | 0 | 0.103 |
| Dexio | 34 | 361 | 0 | 2 | 5 | 0 | 0.102 |
| Noise | 0 | 0 | 38 | 0 | 1 | 0 | 0.026 |
| Para | 34 | 3 | 0 | 332 | 22 | 1 | 0.153 |
| Spath | 7 | 11 | 0 | 45 | 274 | 10 | 0.210 |
| Spiro | 0 | 3 | 0 | 3 | 4 | 339 | 0.029 |
|  |  |  |  |  |  |  |  |
|  | Support Vector Machine | | | | | | |
| Colp | 375 | 1 | 0 | 6 | 5 | 0 | 0.031 |
| Dexio | 41 | 355 | 0 | 2 | 4 | 0 | 0.117 |
| Noise | 0 | 0 | 39 | 0 | 0 | 0 | 0 |
| Para | 33 | 1 | 0 | 348 | 10 | 0 | 0.112 |
| Spath | 5 | 3 | 0 | 42 | 292 | 5 | 0.159 |
| Spiro | 0 | 2 | 0 | 1 | 4 | 342 | 0.020 |

**S3. Species abundances**

**Table S3:** Effects of community composition on the log density of focal species. CI = confidence interval.

|  | *Colpidium* | | | *Dexiostoma* | | | *Paramecium* | | | *Spirostomum* | | | *Spathidium* | | |
| --- | --- | --- | --- | --- | --- | --- | --- | --- | --- | --- | --- | --- | --- | --- | --- |
|  | Beta | 95% CI1 | p-value | Beta | 95% CI1 | p-value | Beta | 95% CI1 | p-value | Beta | 95% CI1 | p-value | Beta | 95% CI1 | p-value |
| Intercept | 5.2 | 5.0, 5.4 | <0.001 | 5.7 | 5.5, 5.9 | <0.001 | 4.9 | 4.7, 5.1 | <0.001 | 3.8 | 3.6, 3.9 | <0.001 | 2.5 | 2.4, 2.6 | <0.001 |
| Predation | -1.9 | -2.1, -1.6 | <0.001 | -1.8 | -2.0, -1.5 | <0.001 | -1.5 | -1.7, -1.2 | <0.001 | -1.7 | -1.9, -1.5 | <0.001 | - | - | - |
| *Paramecium* present | -0.60 | -0.89, -0.32 | <0.001 | -0.81 | -1.1, -0.57 | <0.001 | - | - | - | - | - | - | 0.66 | 0.48, 0.84 | <0.001 |
| *Spirostomum* present | 0.06 | -0.22, 0.34 | 0.7 | -0.43 | -0.67, -0.19 | 0.001 | - | - | - | - | - | - | -0.17 | -0.36, 0.01 | 0.062 |
| Predation and *Paramecium* present | 0.83 | 0.43, 1.2 | <0.001 | 0.96 | 0.62, 1.3 | <0.001 | - | - | - | - | - | - | - | - | - |
| Predation and *Spirostomum* present | -0.04 | -0.44, 0.36 | 0.8 | 0.24 | -0.10, 0.58 | 0.2 | - | - | - | - | - | - | - | - | - |

**S4: Model fitting to estimate interactions**

The following section shows the coefficient estimates for the additive and interactive Lotka-Volterra and Ricker competition models.
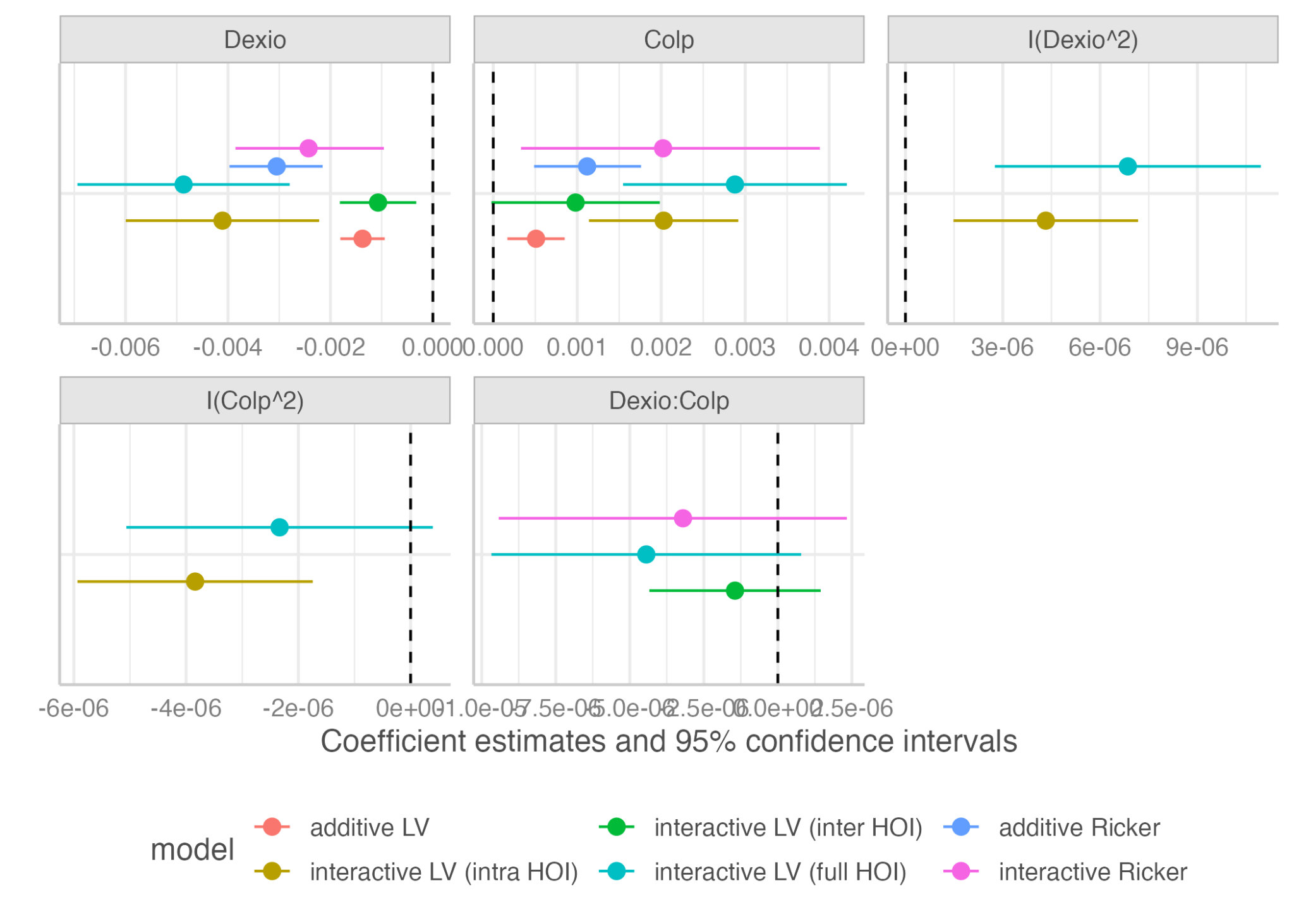


Figure S1: *Dexiostoma* population growth rate in CD community


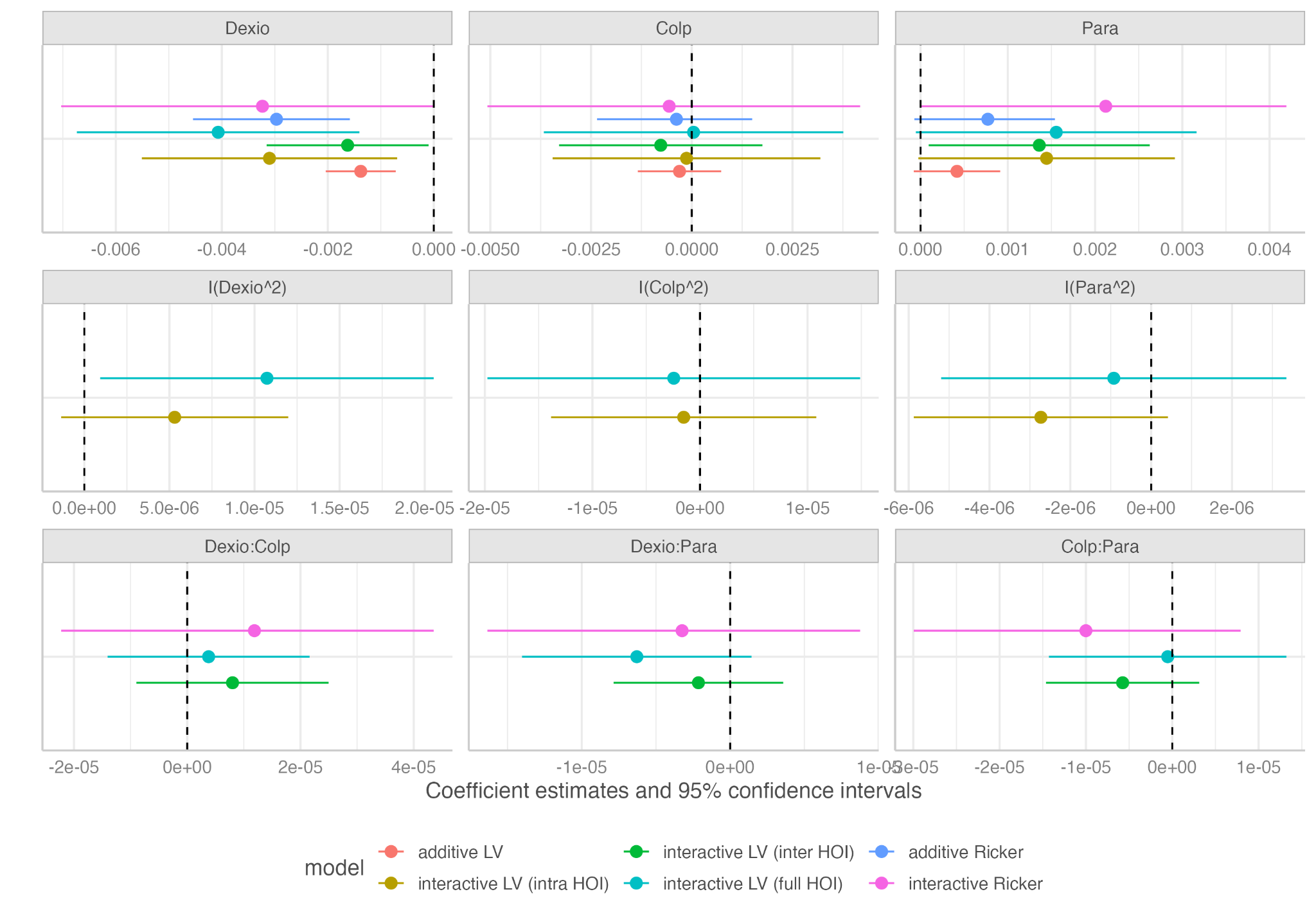
Figure S2: *Dexiostoma* population growth rate in CDP community


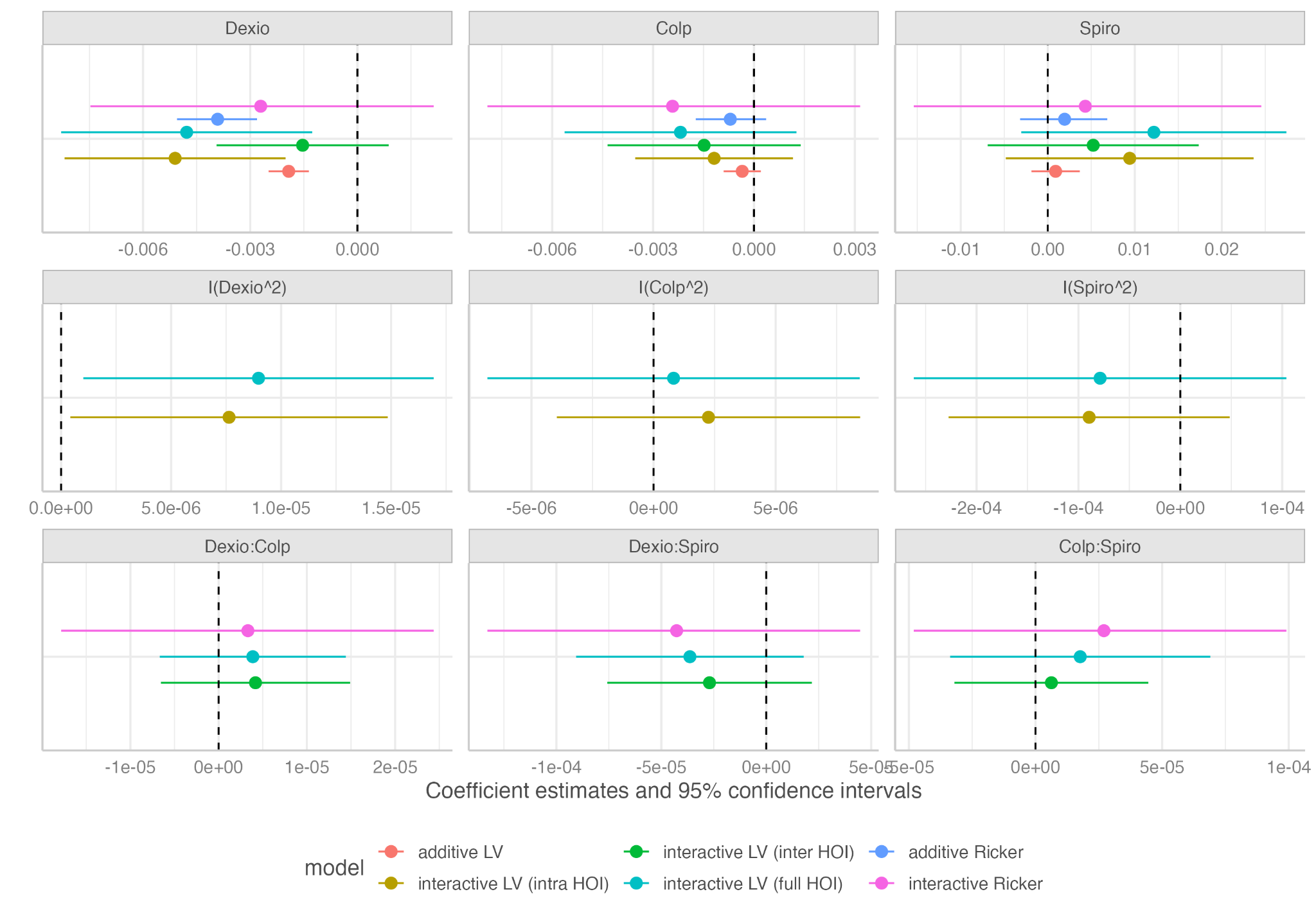


Figure S3: *Dexiostoma* population growth rate in CDS community


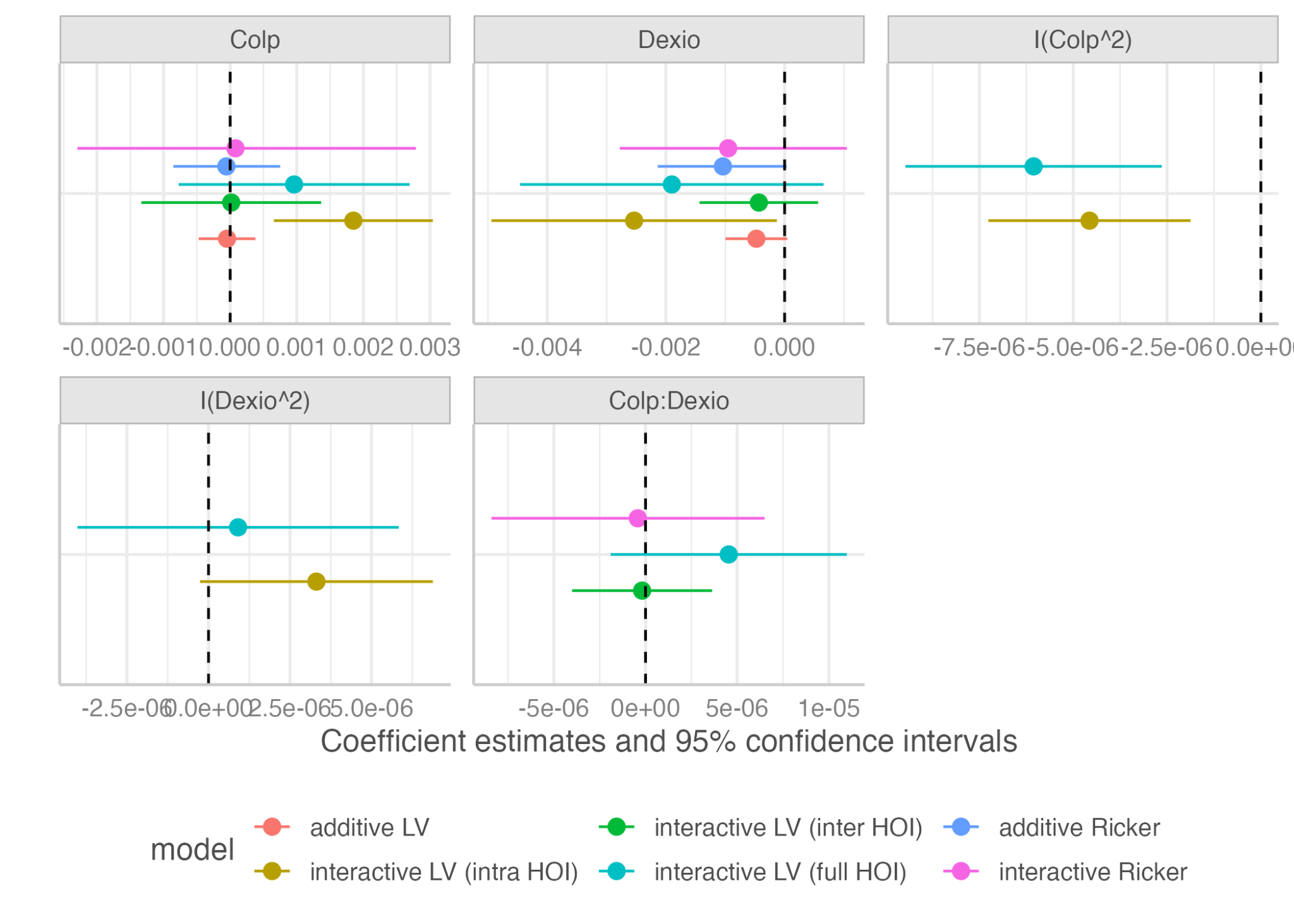


Figure S4: *Colpidium* population growth rate in CD community


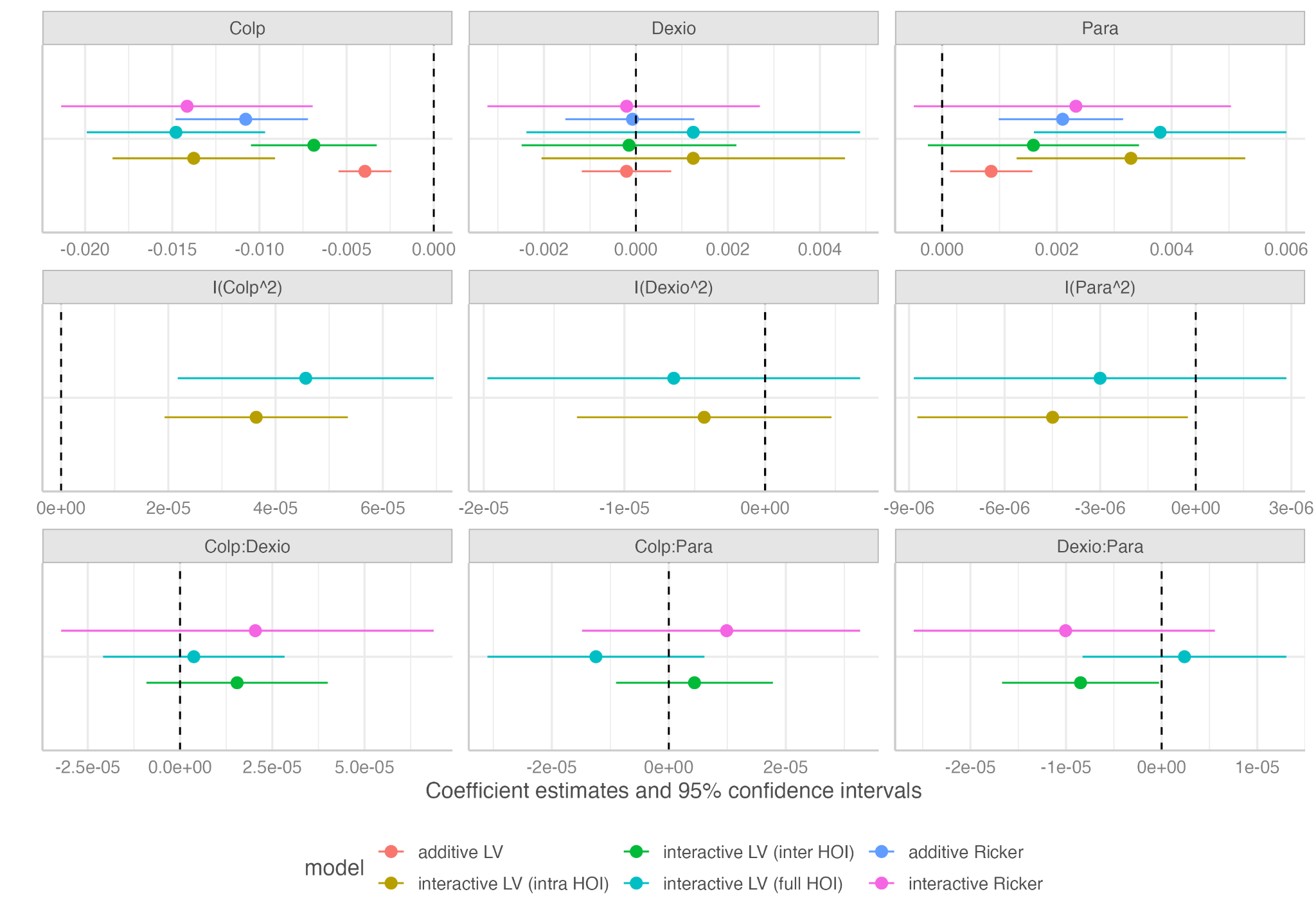


Figure S5: *Colpidium* population growth rate in CDP community


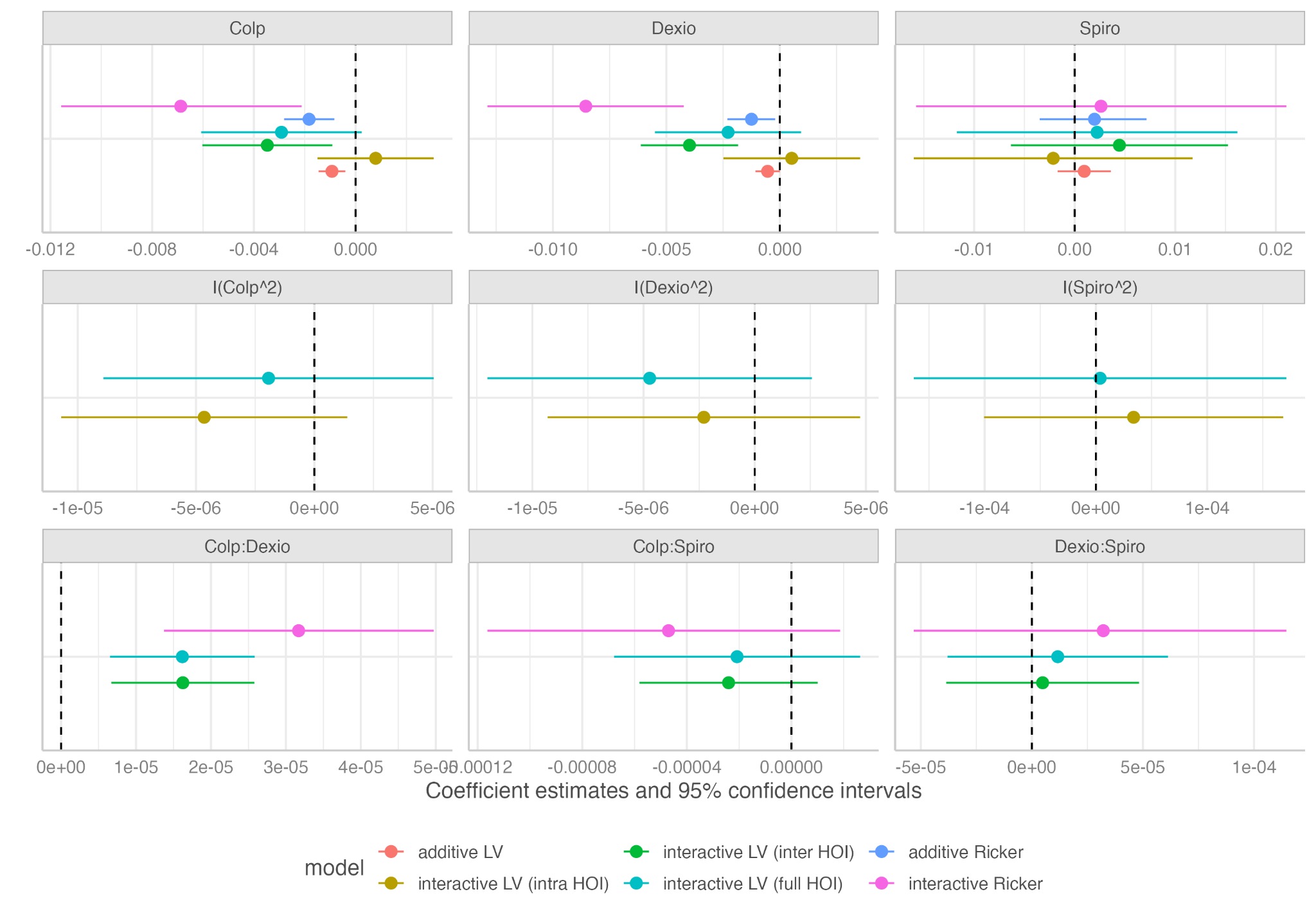


Figure S6: *Colpidium* population growth rate in CDS community


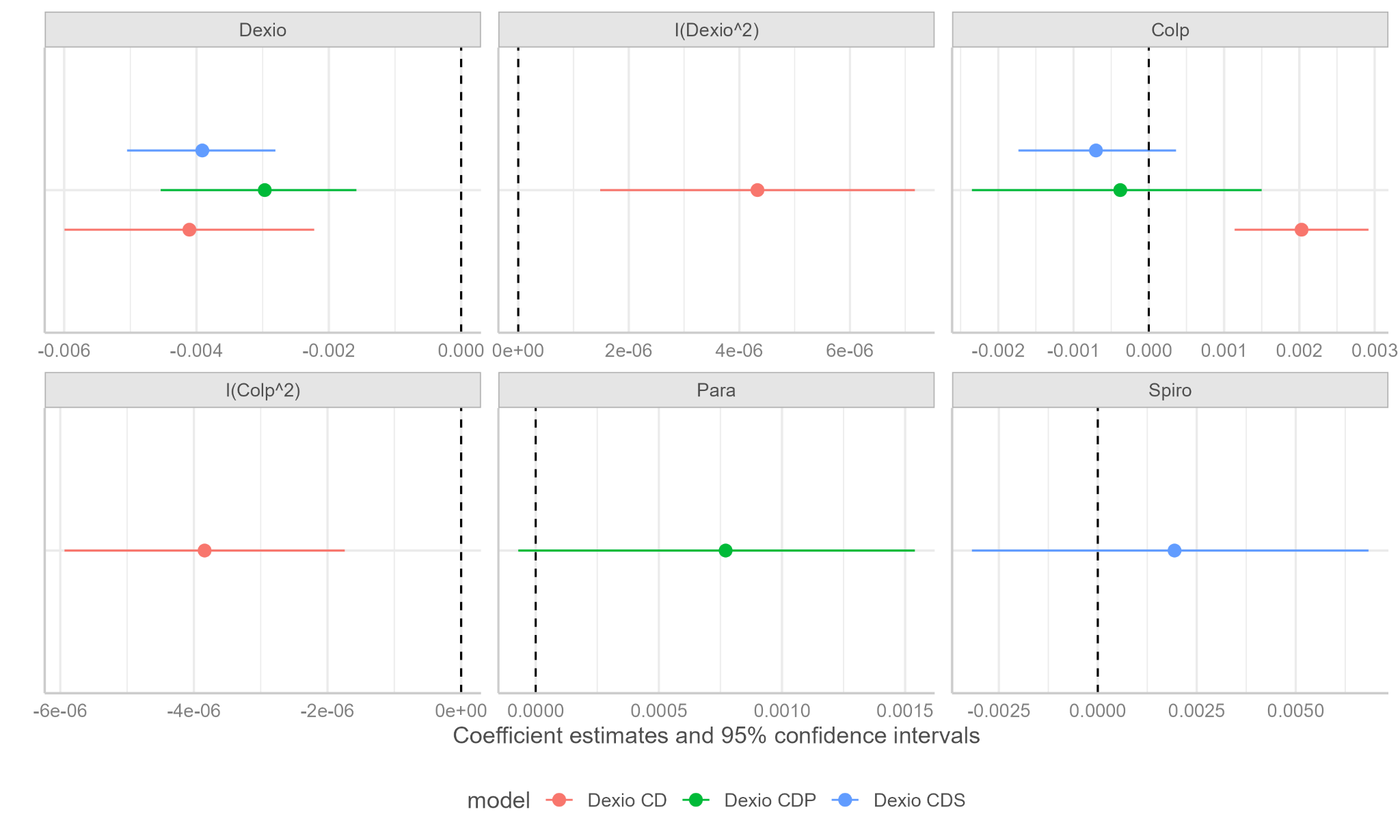


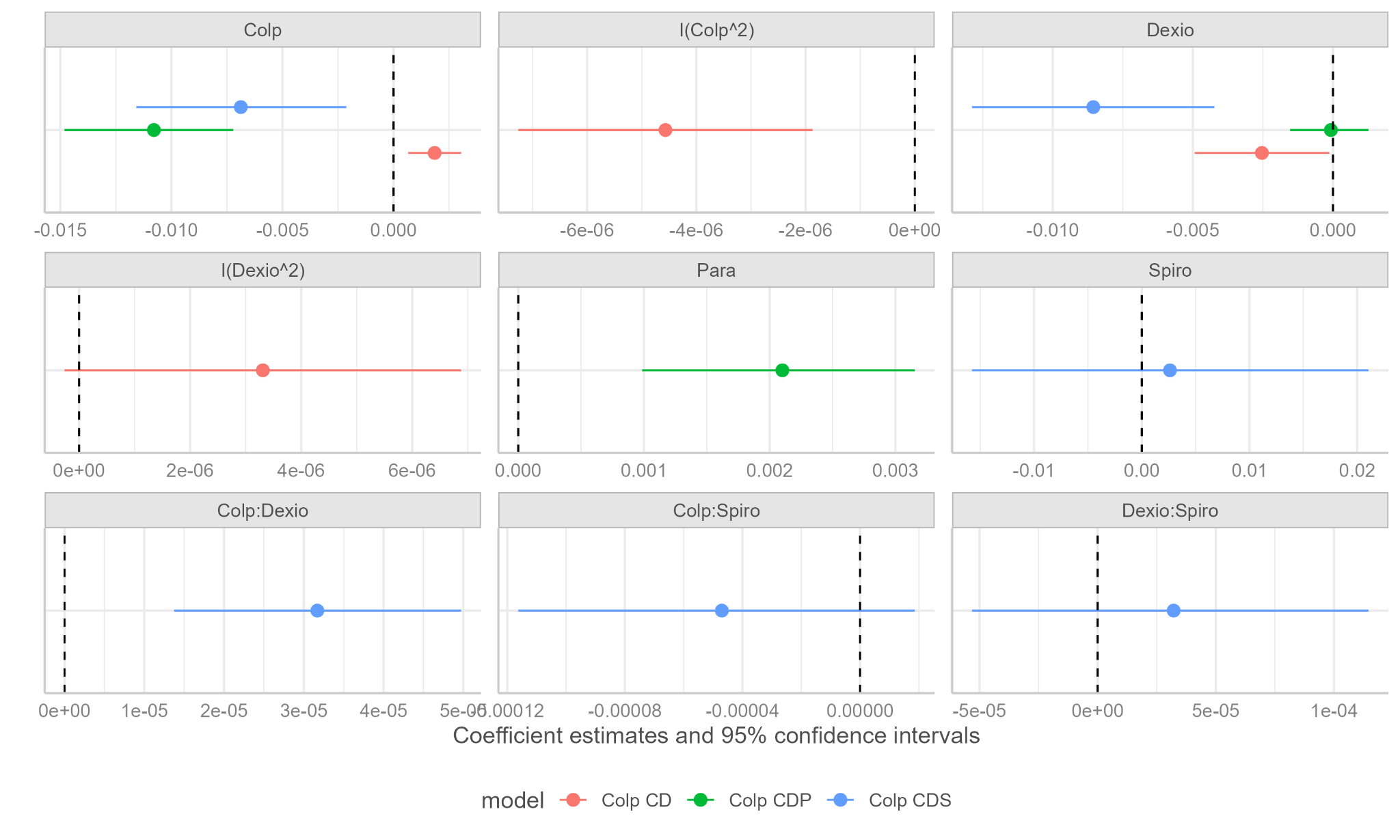
Figure S7: Coefficient plots for the best supported models for *Dexiostoma* (upper panel) and *Colpidium* (lower panel) across community compositions. Each panel shows the intra- or interspecific interaction terms as well as the interaction coefficients.

**S5. Model fit:**

We show the fit of the most parsimonious model compared to the additive LV model first for Colpidium, then for Dexiostoma, across all community compositions. The Y shows the population growth rate, whereas the x-axis shows the effects of intra- or interspecific density. Changes in model fit across panels show intra- or interspecific higher order interactions.


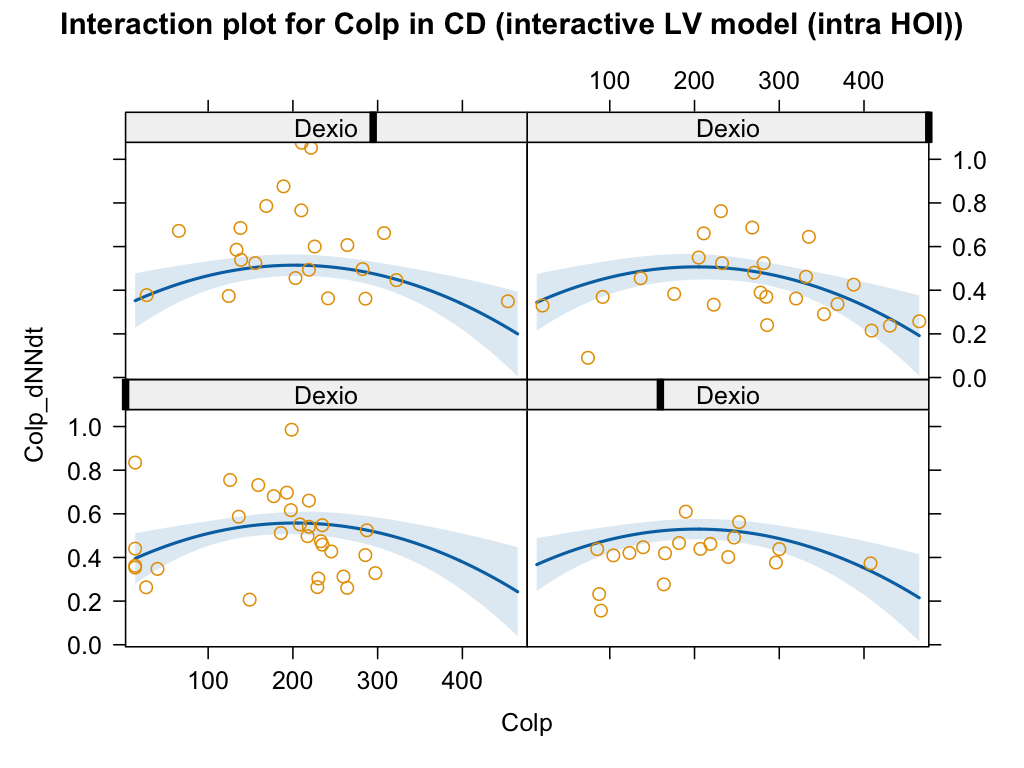

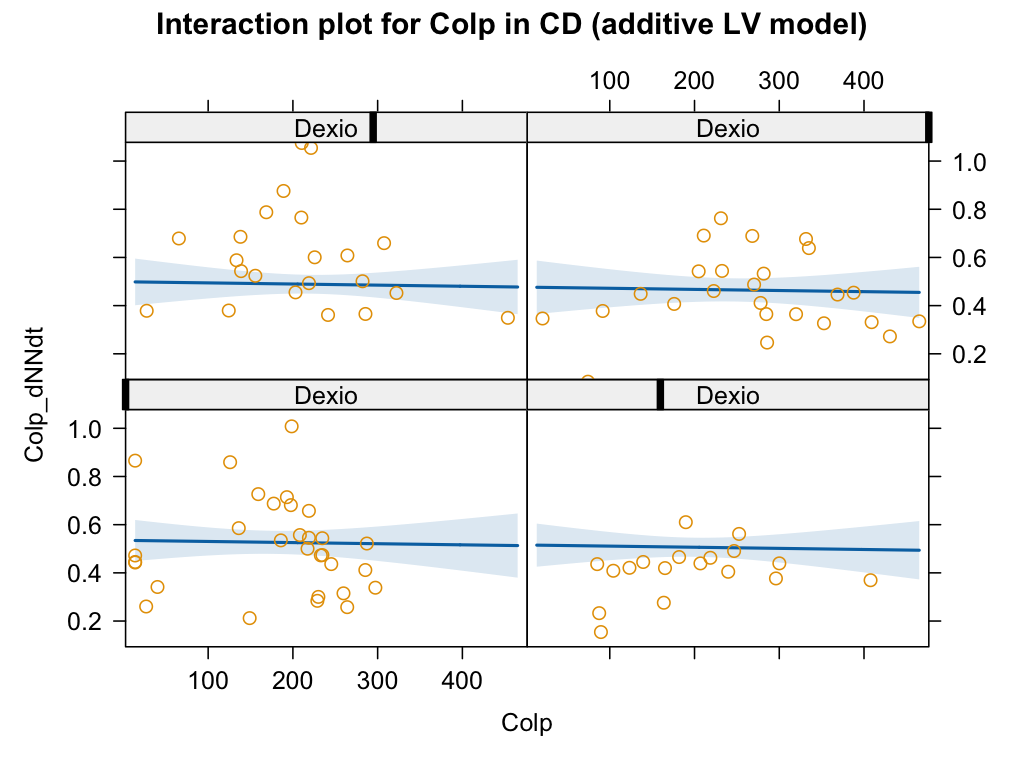


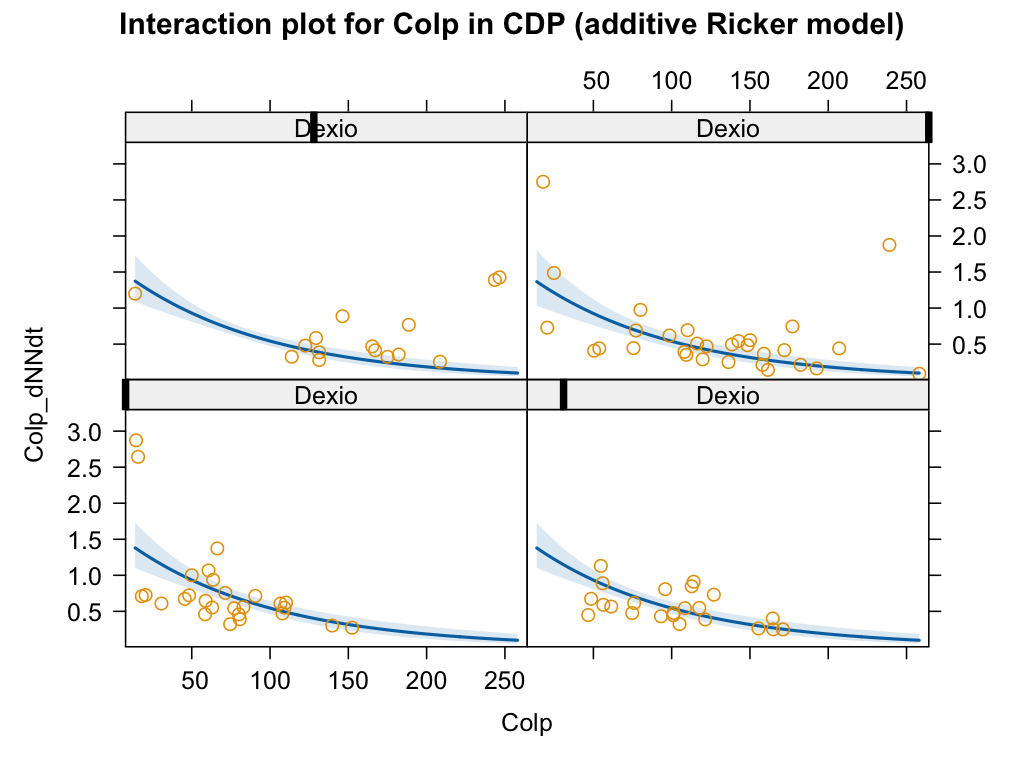

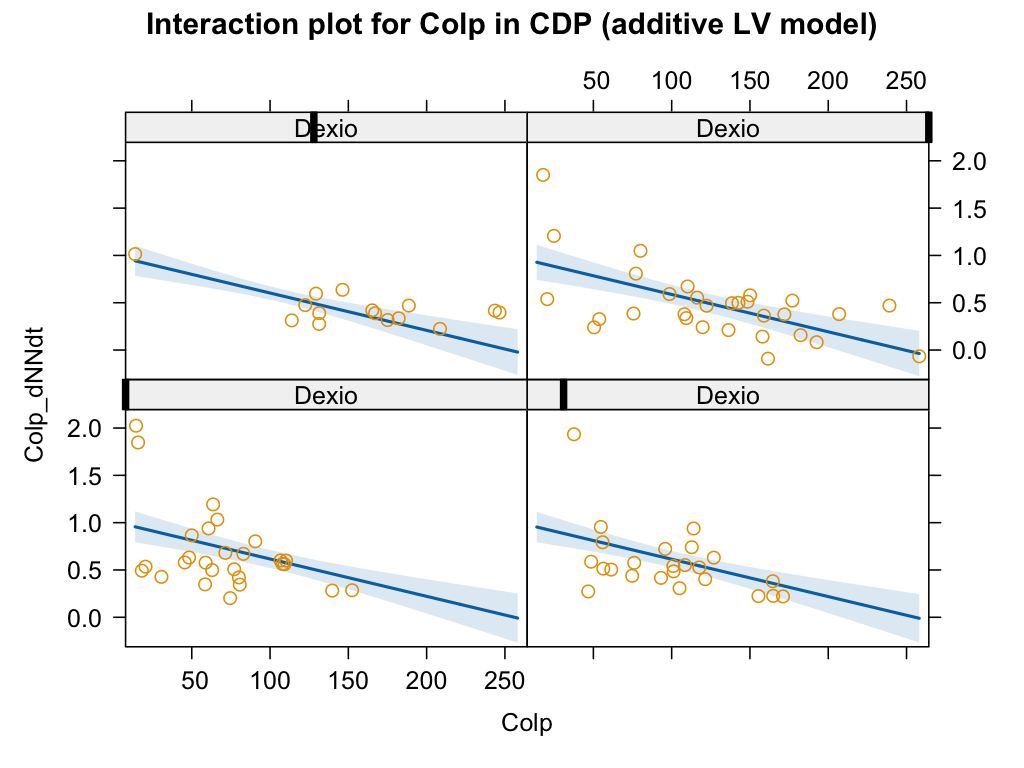

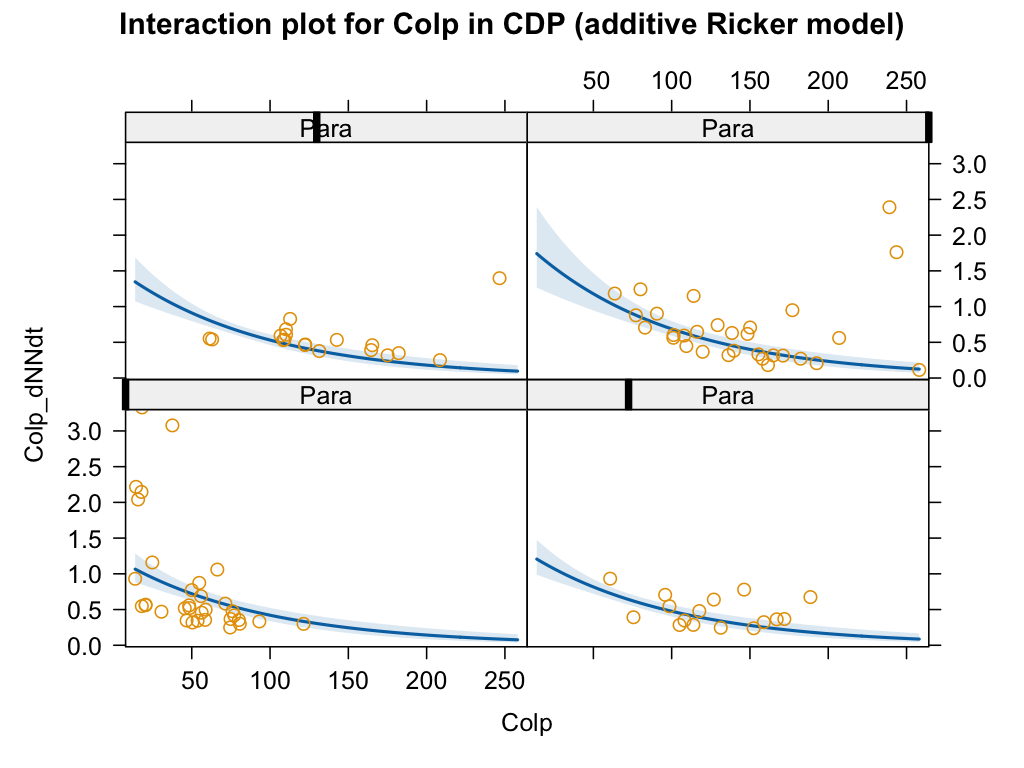

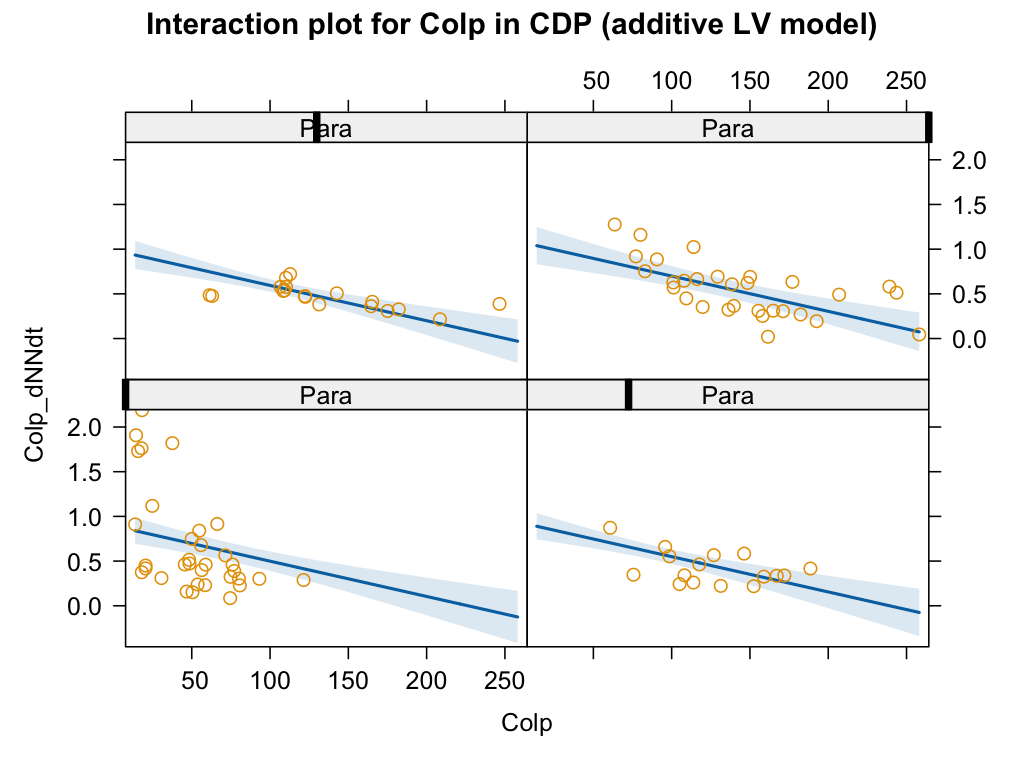

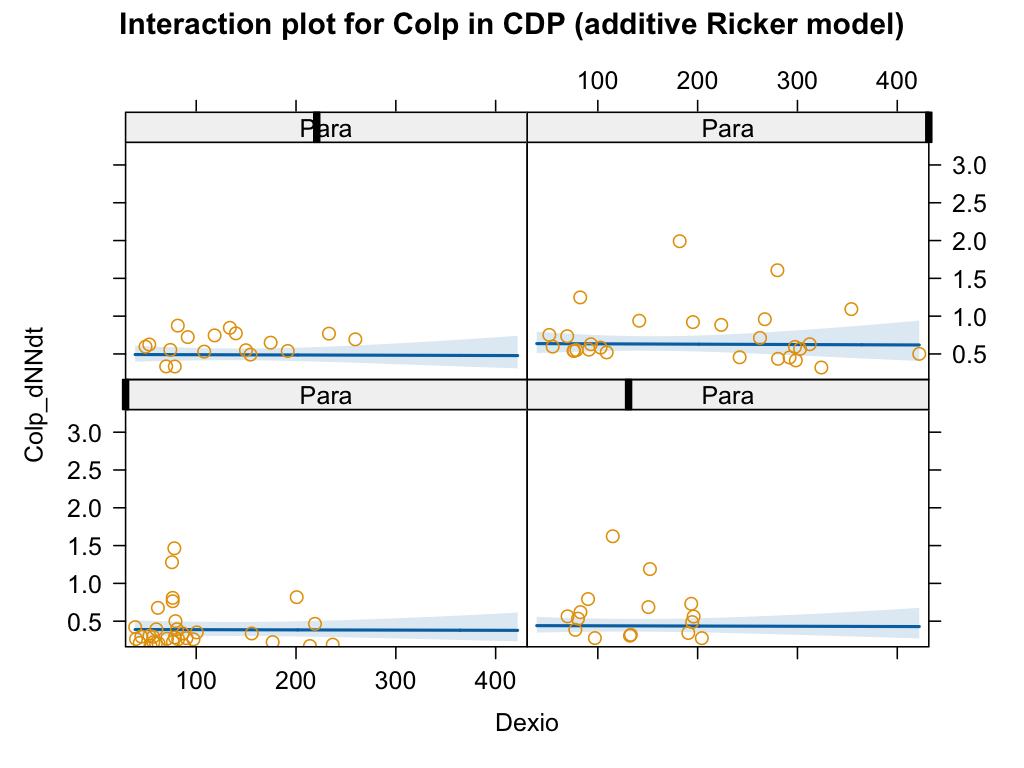

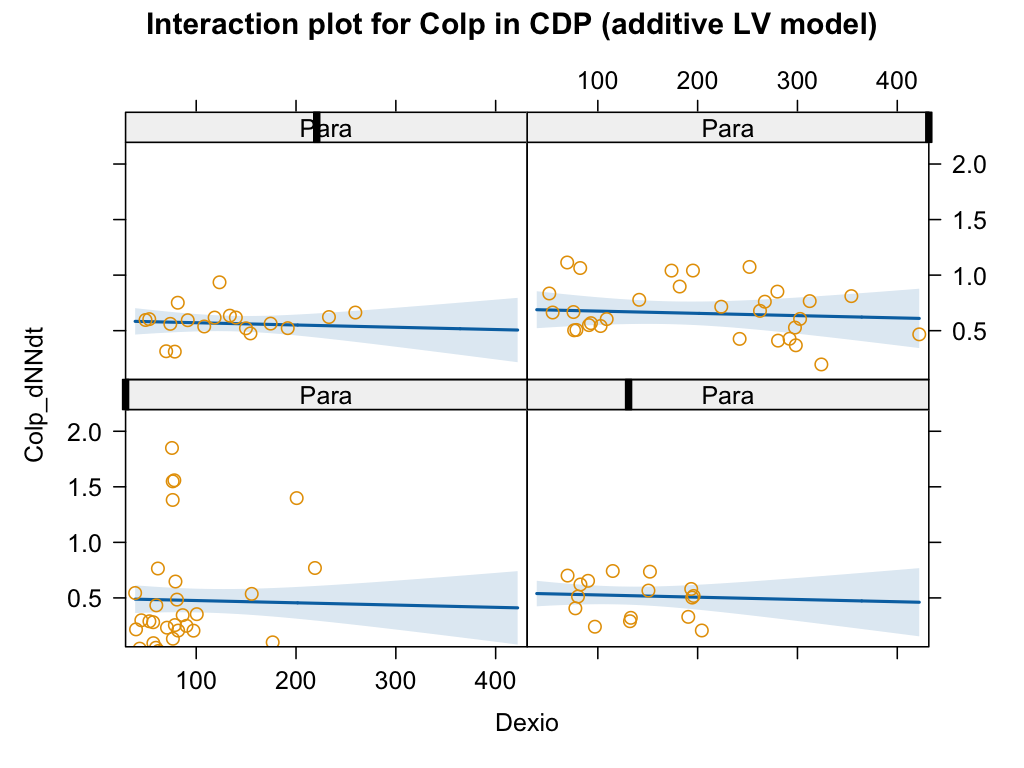

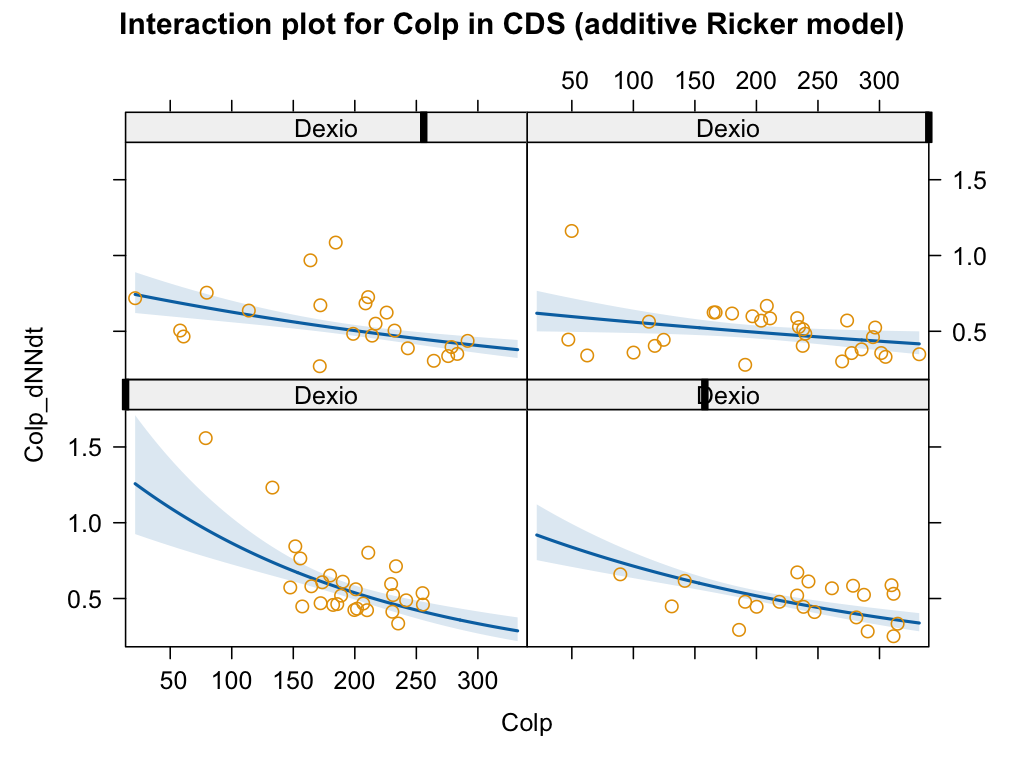

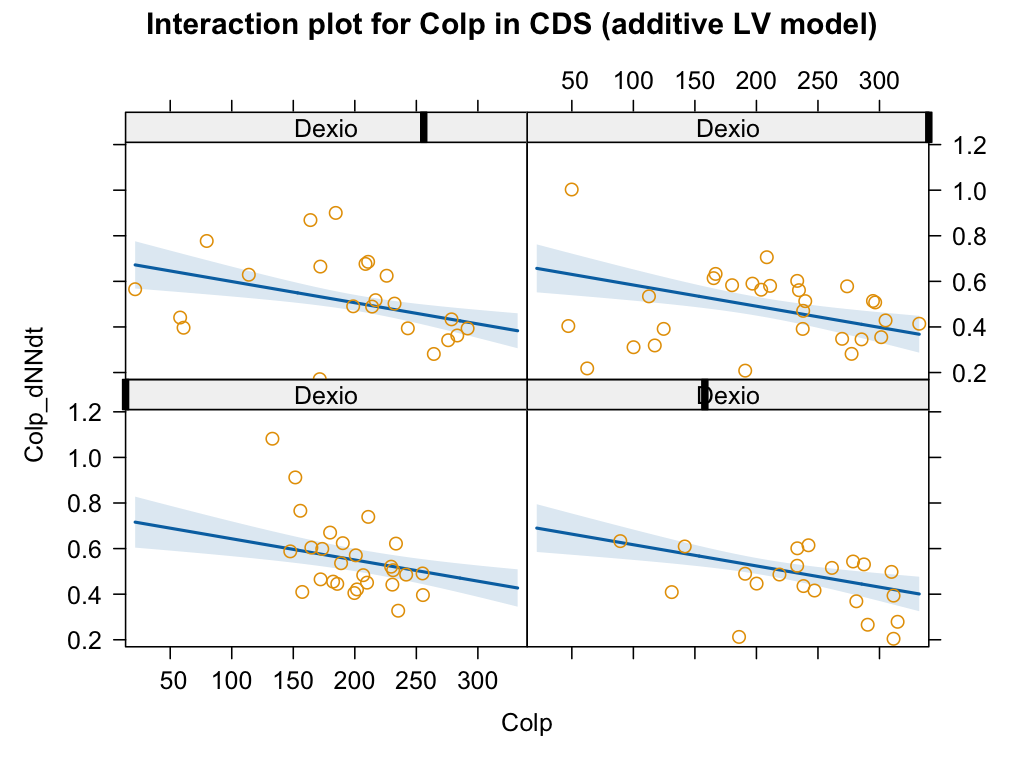

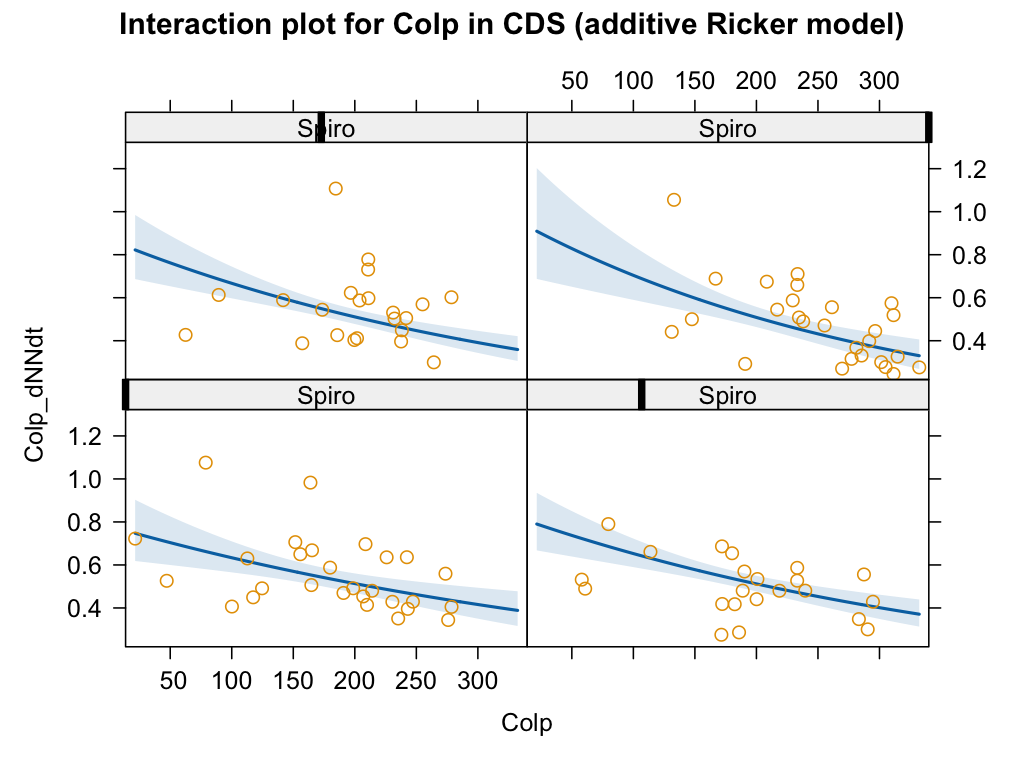

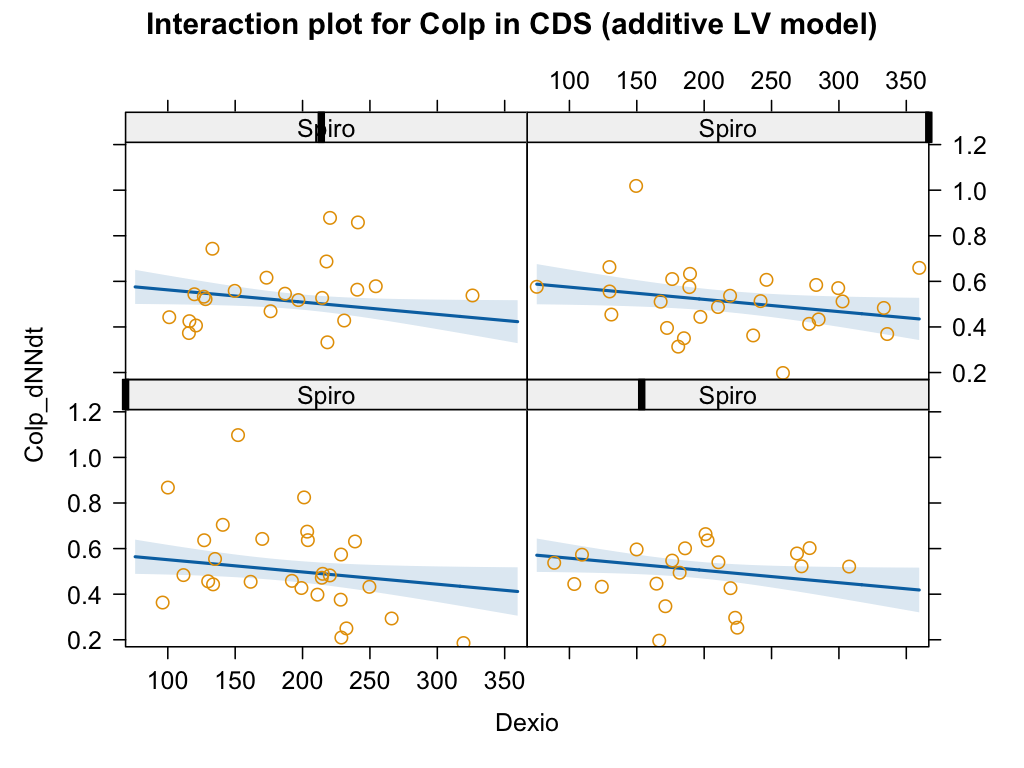

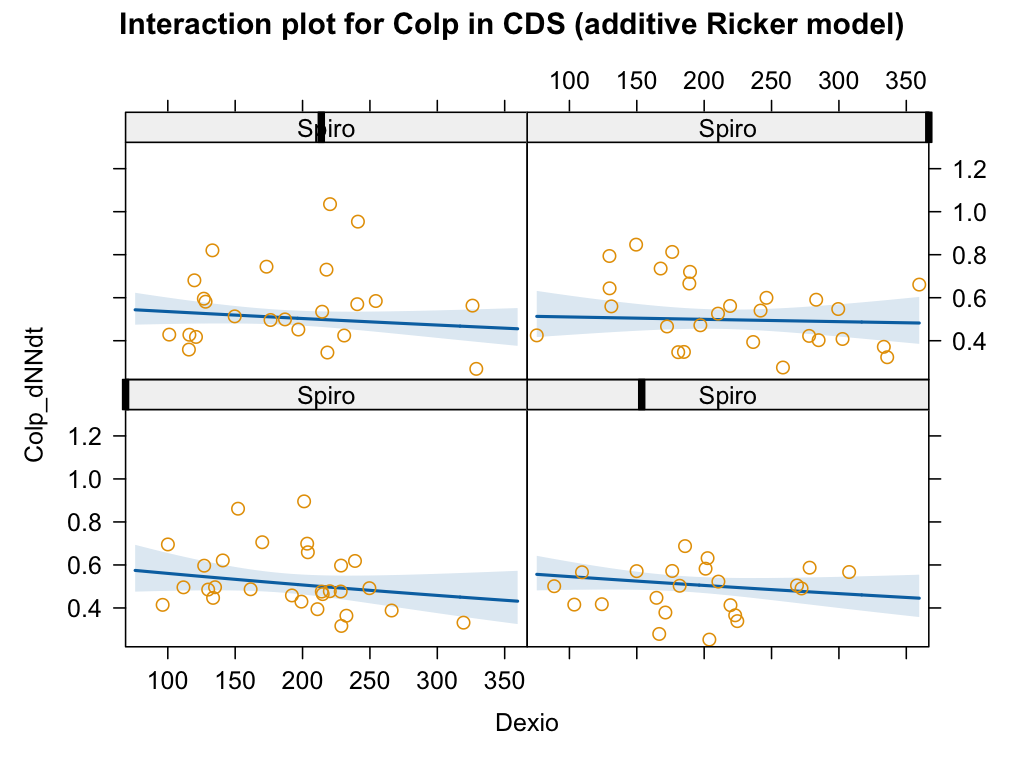

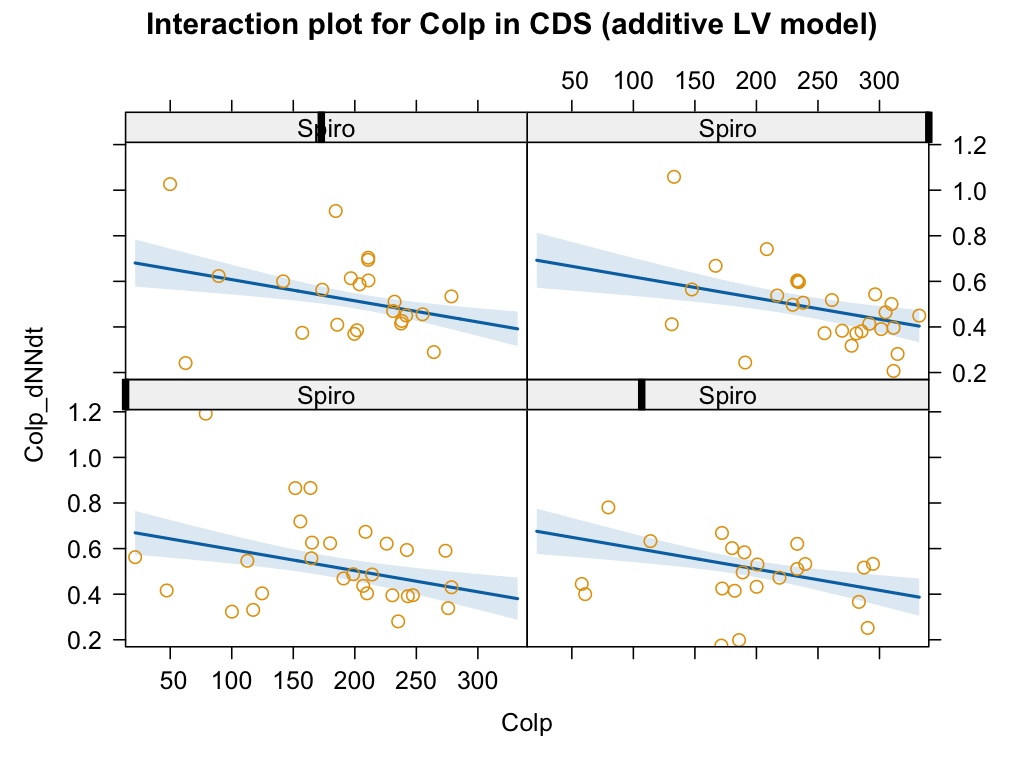

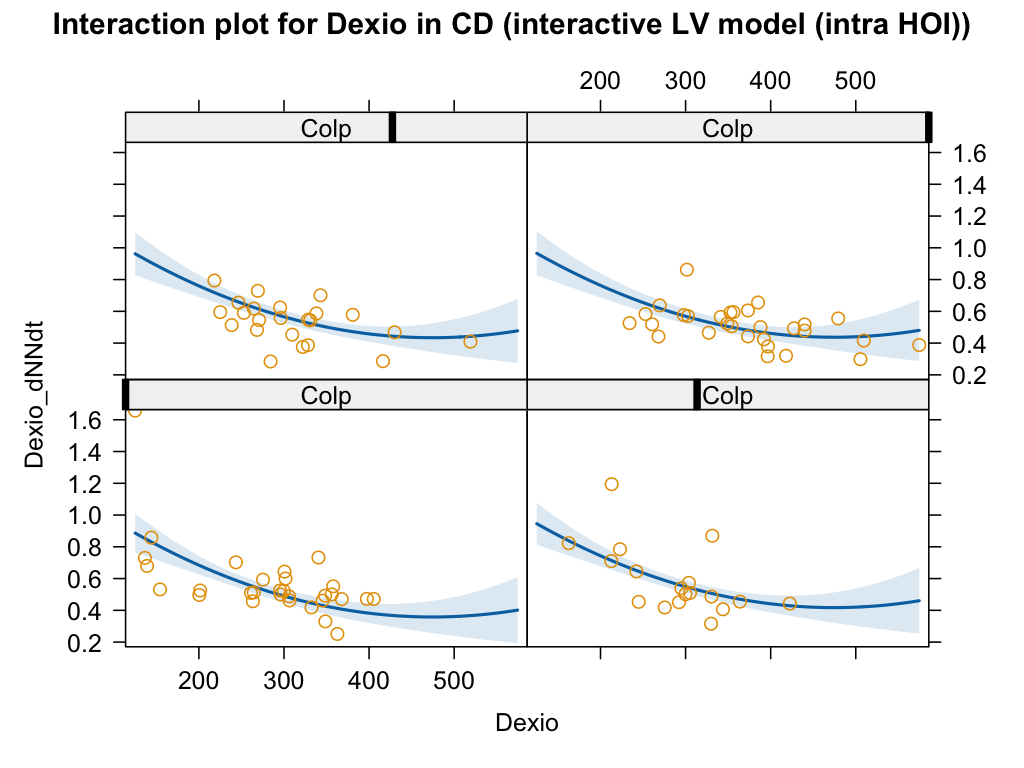

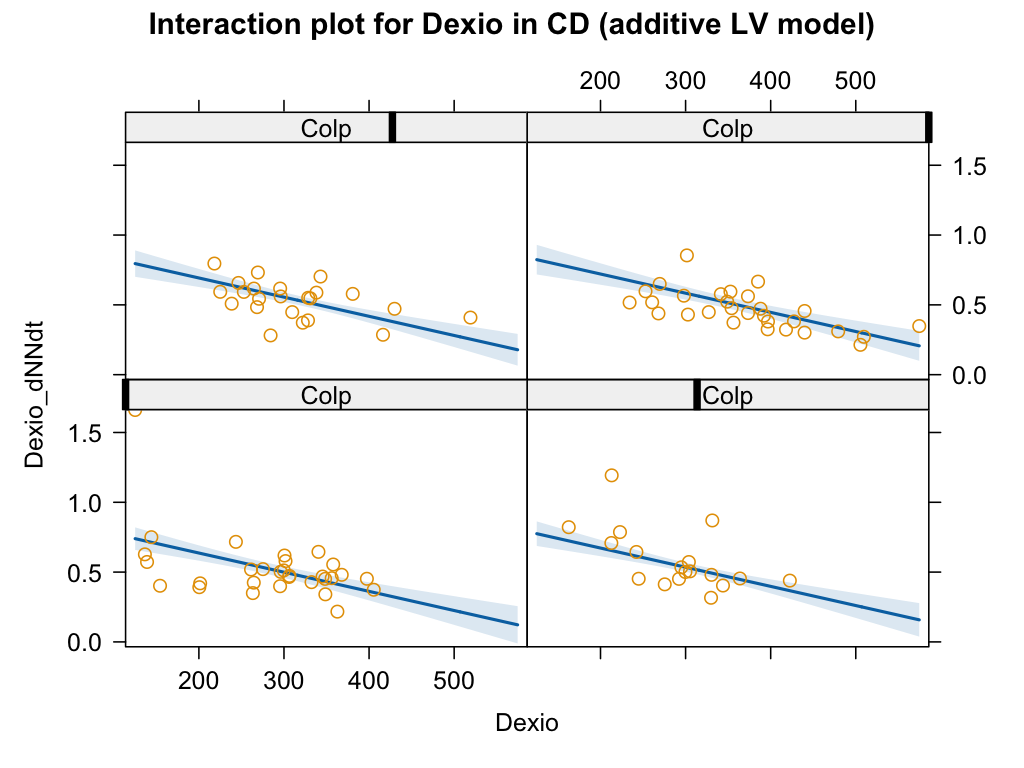

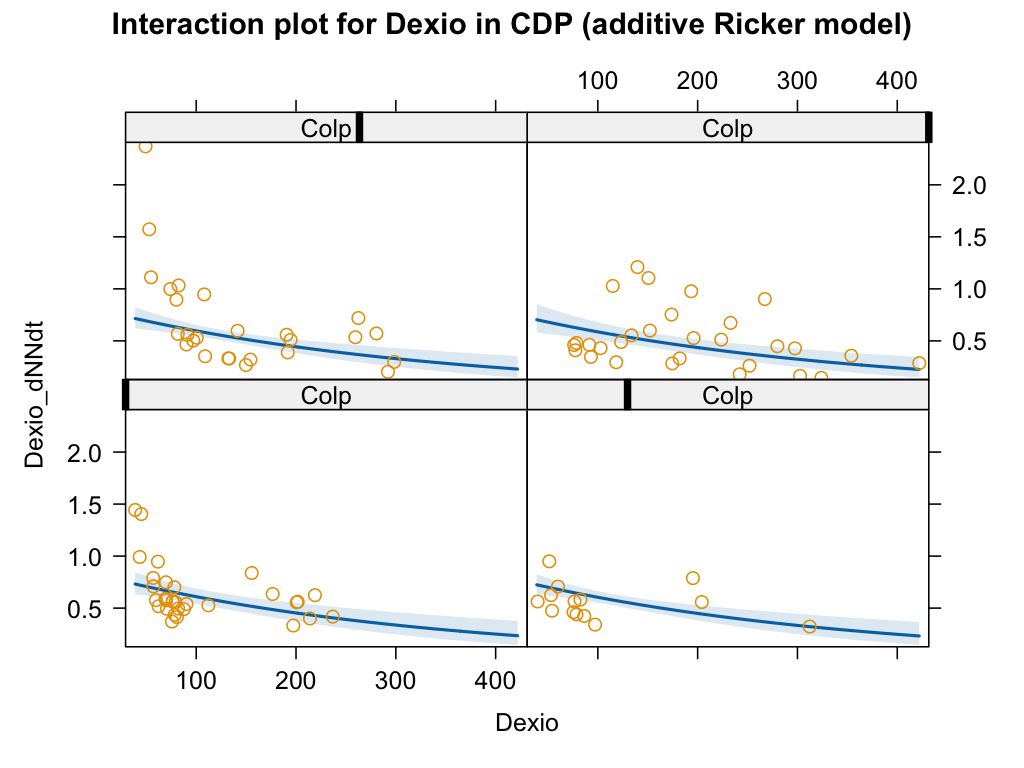

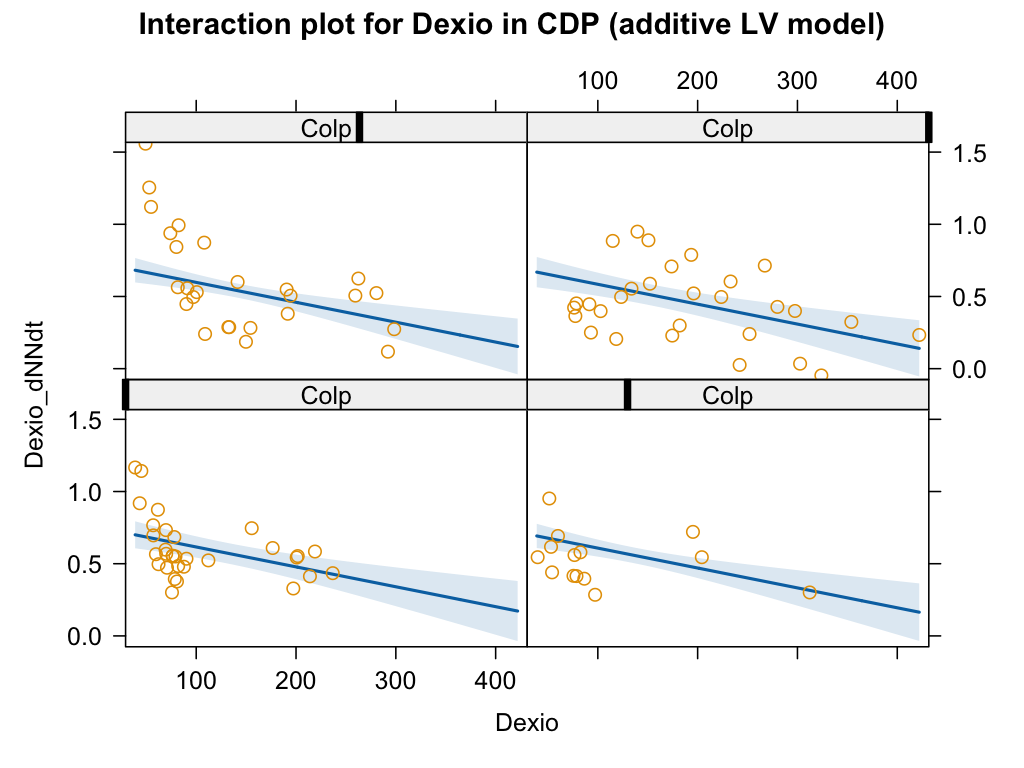

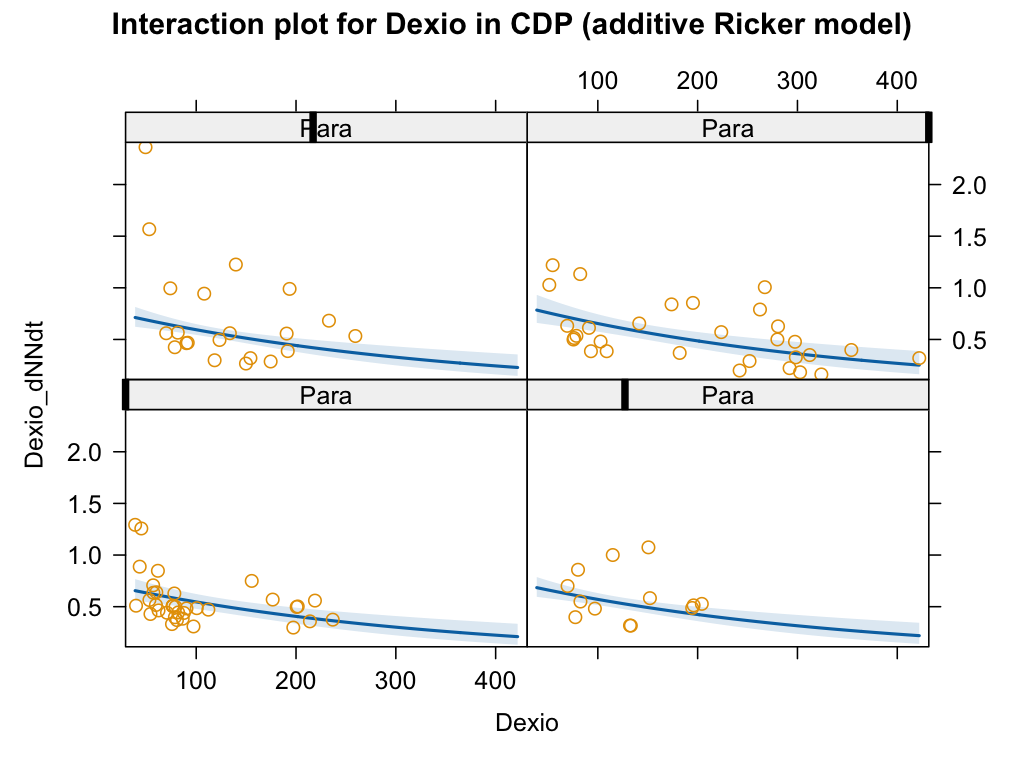

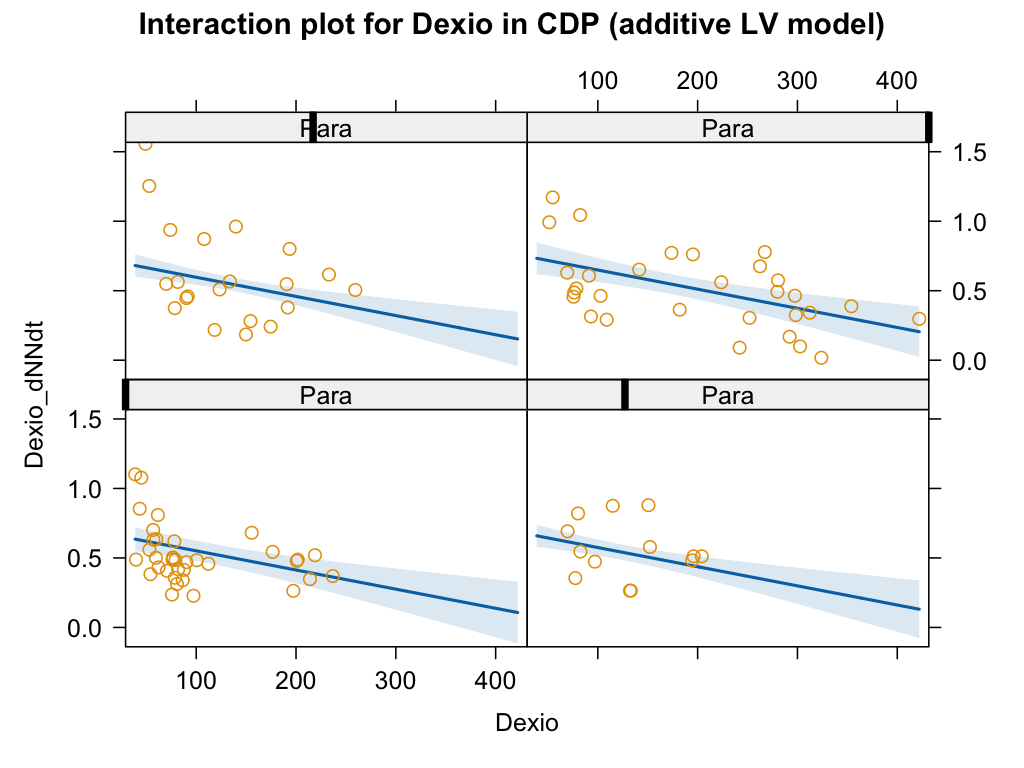

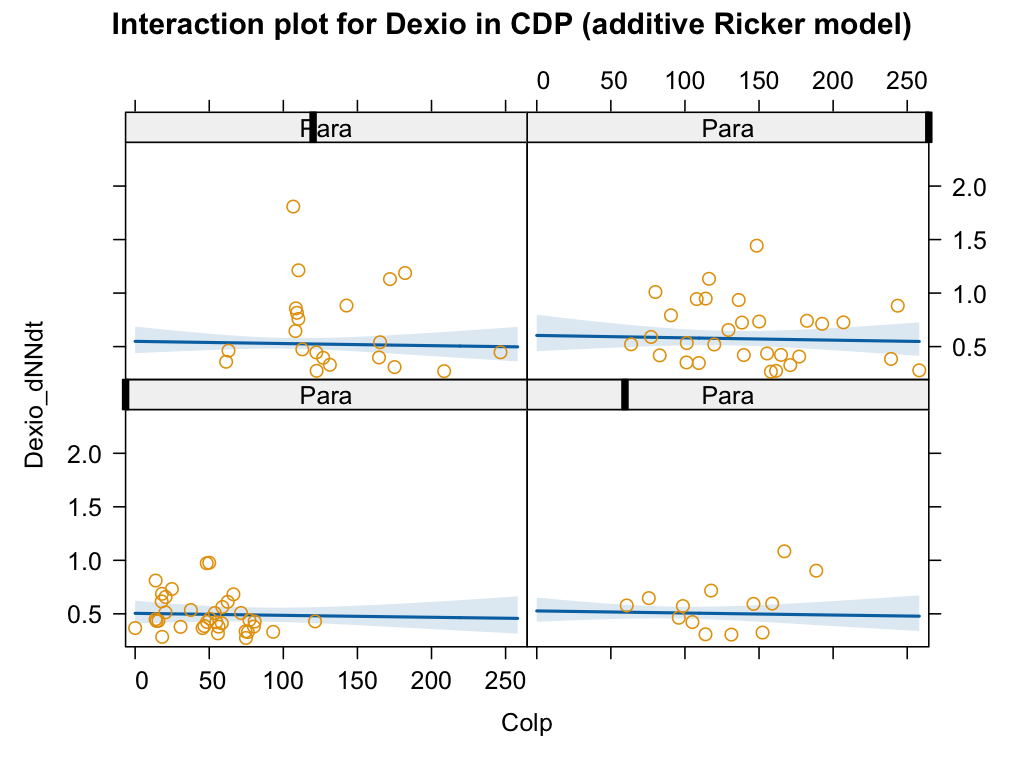

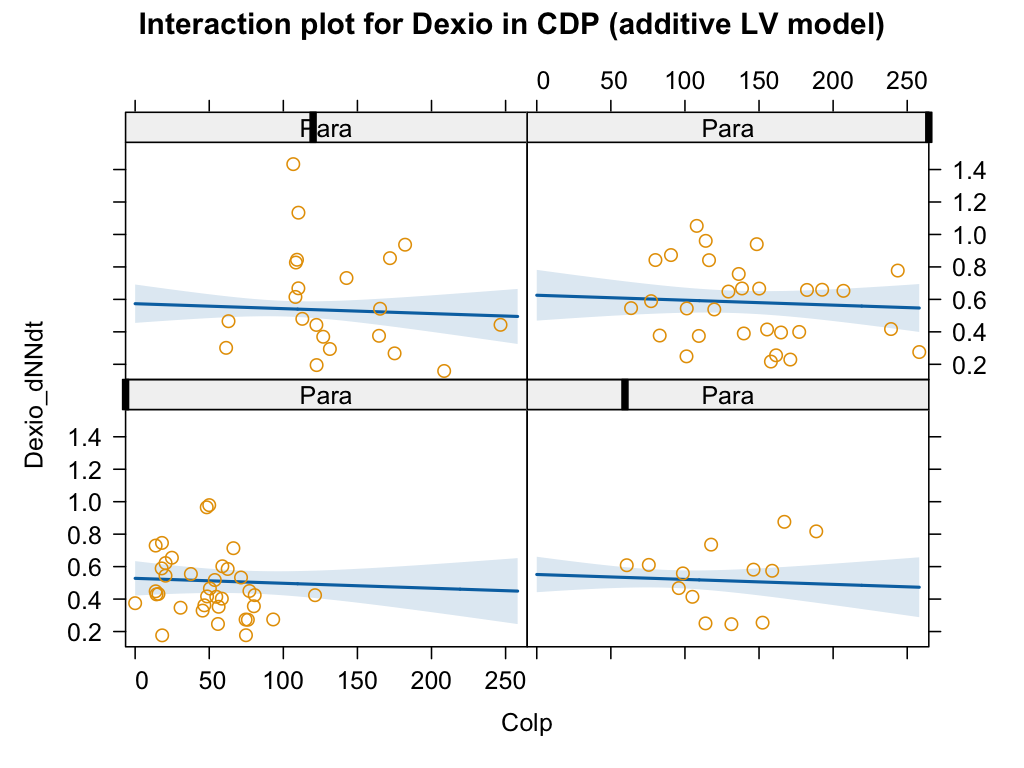

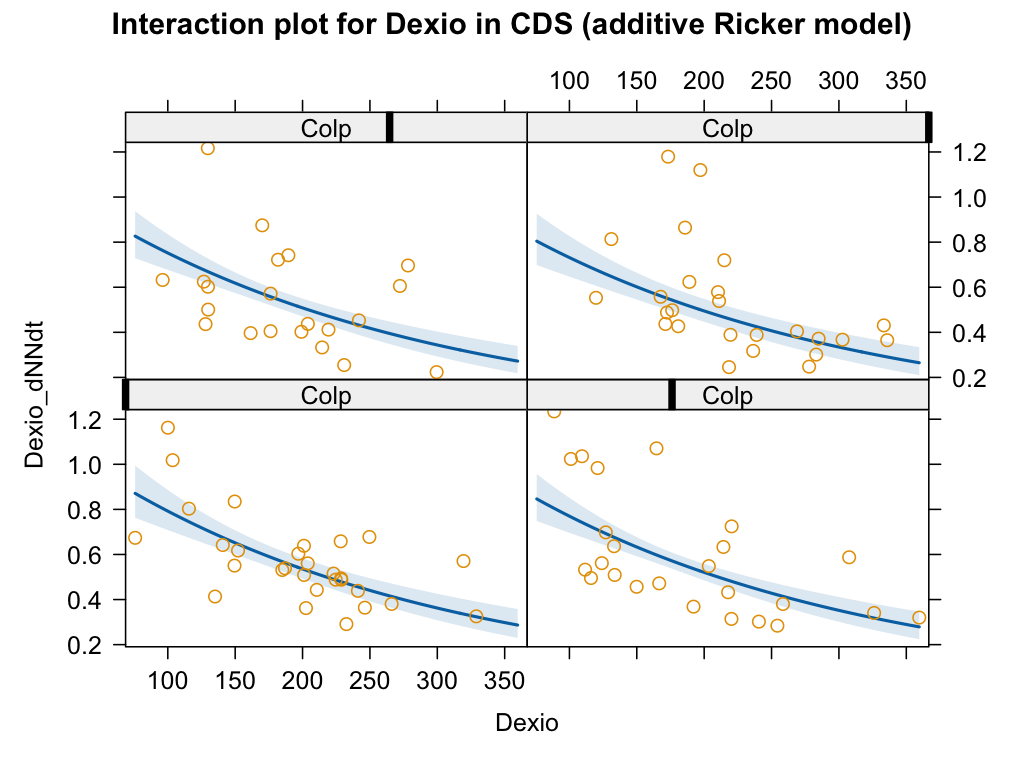

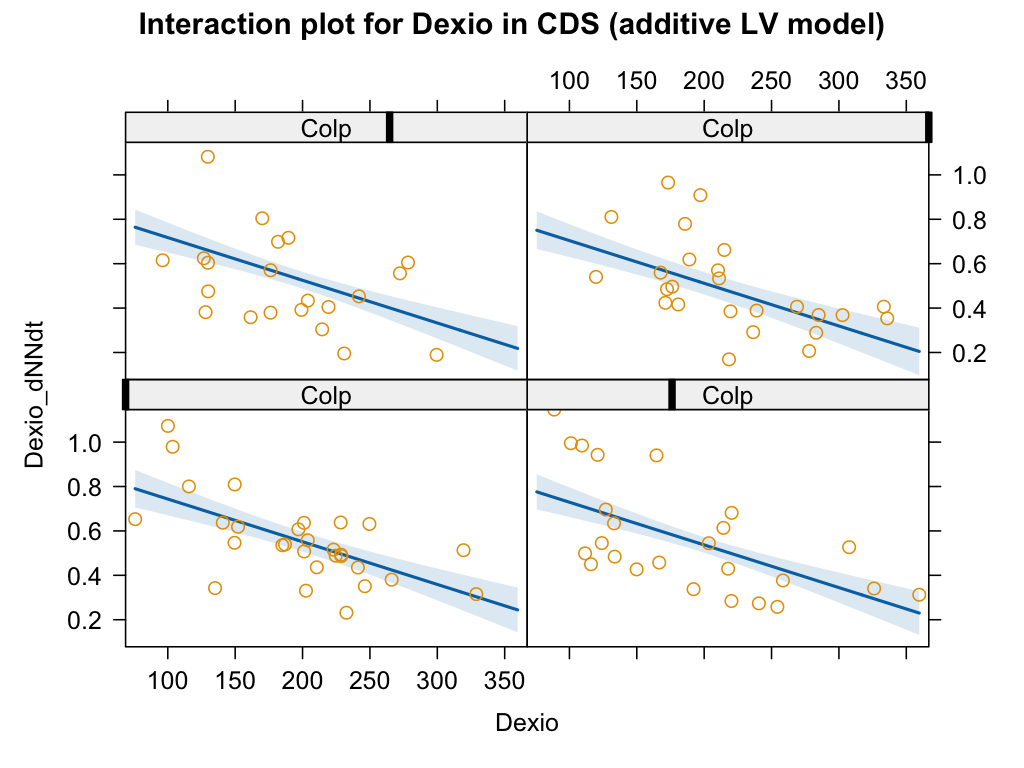

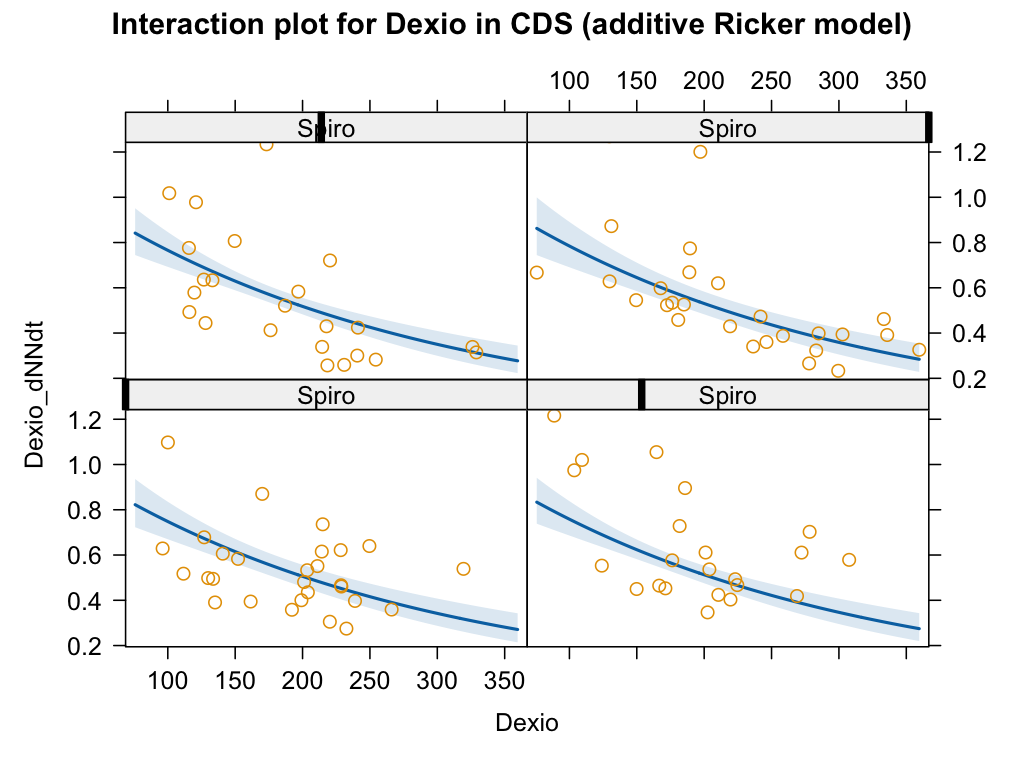

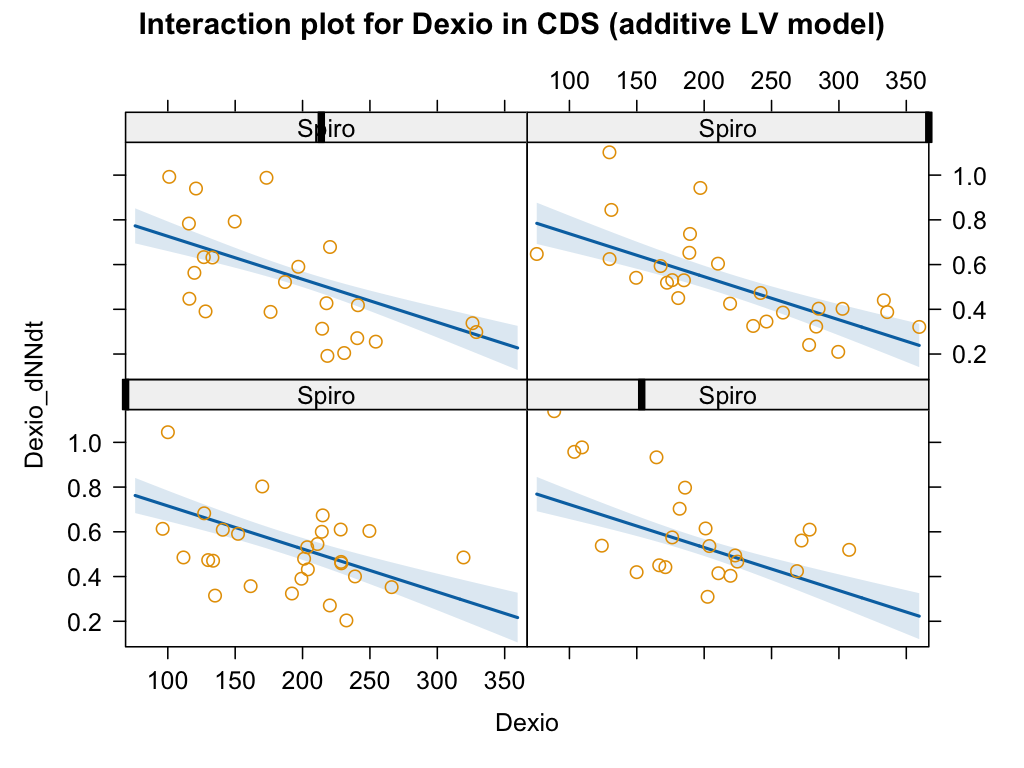

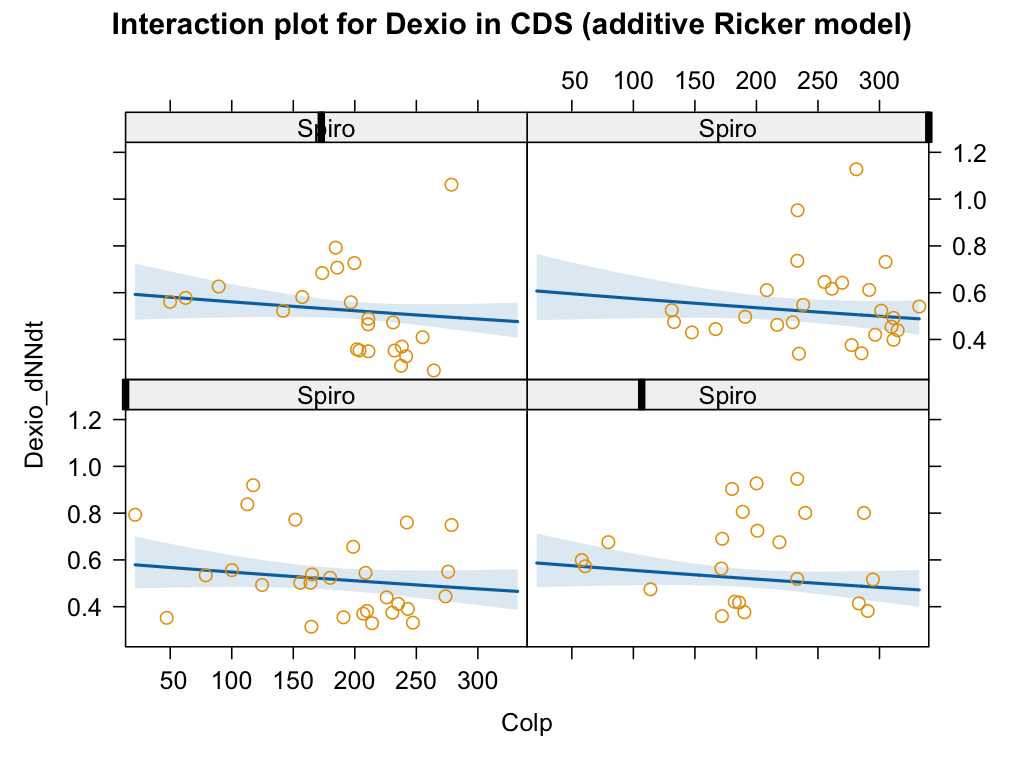

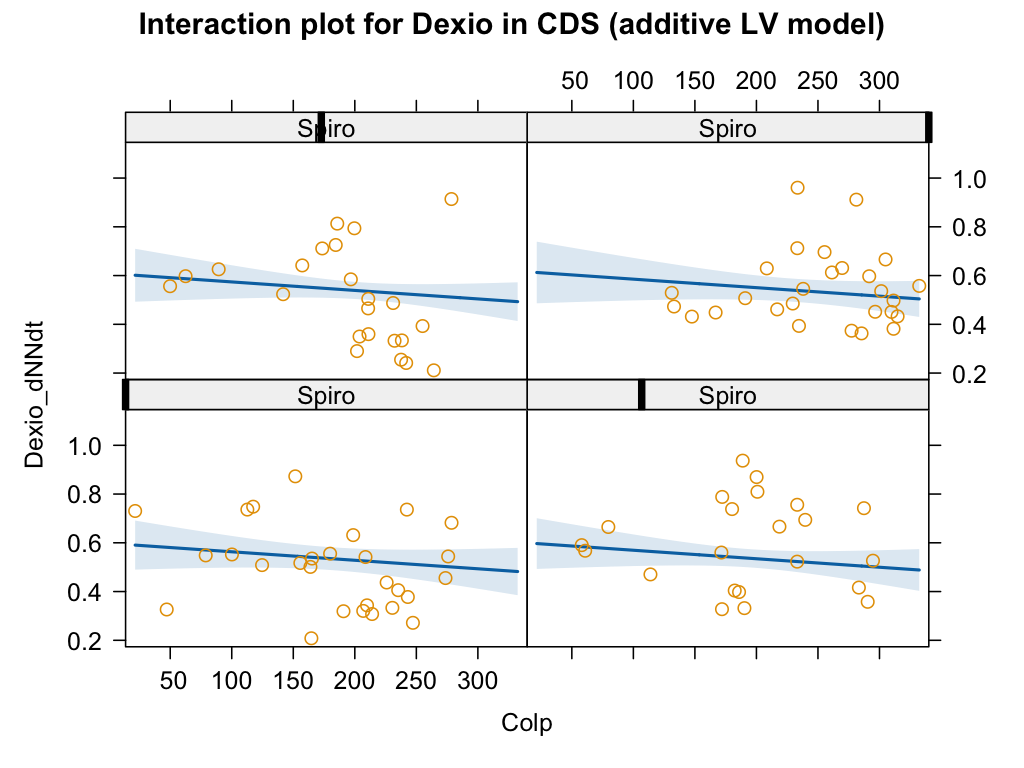

Supplement: Supplementary file 1 — Appendix S1 [file ECE3-13-e10502-s001.docx]
